# Ebola: is the response justified?

**DOI:** 10.11694/pamj.supp.2015.22.1.6598

**Published:** 2015-10-11

**Authors:** Hannah Lewis, Aisha Chaudry, Gibril Ndow, Mary ME Crossey, Debbie Garside, Ramou Njie, Simon D Taylor-Robinson

**Affiliations:** 1 Medical Education Research Unit, Imperial College London, SW7 2AZ, United Kingdom; 2 MRC Unit The Gambia, Atlantic Road, Fajara, The Gambia, Gambia; 3 Department of Medicine, St Mary's Hospital campus, Imperial College London, W2 1NY, United Kingdom

**Keywords:** Ebola, outbreak, response

## Abstract

Ebola virus disease is a viral hemorrhagic fever, first discovered in 1976 in Sudan, where the outbreak infected over 284 people with a 53% case fatality ratio. There have been 34 further epidemics, the current major incident in West Africa having recorded more cases and deaths than all previous outbreaks combined. To date there have been over 27, 000 confirmed, probable and suspected cases and 11,000 reported deaths in Liberia, Guinea and Sierra Leone. With total funding and pledges to help control the outbreak amounting to more than US$2.4billion, many question how the disease has continued to spread in Sierra Leone and Guinea Conakry, and whether the response to the outbreak has been justified. This article aims to analyze the effectiveness of the responses to the outbreak in terms of economic, social, cultural and, to an extent, political impact. We argue that the response has been justified due to the awareness raised, the infrastructure and staffing improvements, the success in receiving financial aid and the minimal spread to other countries outside the main transmission zone. Despite this, some failures in communication and a slow early response were noted.

## Introduction

The Ebola Virus Disease (EVD) outbreak emerged in a remote corner of Guinea in December 2013, and spread into Liberia and Sierra Leone in the context of weak health systems in all three countries [1, 2]. To date, around ten thousand people have died from the worst outbreak of the disease on record. At the end of November 2014, case incidence was still increasing in Guinea and Sierra Leone while stabilising or declining in Liberia. In Guinea, 75 to 148 confirmed cases were being reported on a weekly basis between October and end of November. By 1st December 17 districts out of 38 had been affected by the outbreak and more new cases reported in November with an increase of 36% compared to October 2014. In total 2,164 Ebola cases and 1,327 deaths were notified by the Ministry of Health in the beginning of December 2014 [3, 4].

In most of the affected areas interventions like case contact tracing and follow-up, referrals to a health centre when a case is suspected, safe burial practices and disease surveillance at community level were not routinely carried out. There was an insufficient number of case management centres despite the daily increasing demand. The already fragile National Health System in Guinea was rapidly overwhelmed leading to the inevitable accelerated spread of Ebola [1, 2, 5].

The situation required the implementation of immediate and vigorous additional interventions to stop the spread of the epidemic. International aid and support increased and many organizations and institutions joined the response with several projects implemented on the ground. Despite this, the disease continued to spread rapidly in the country. Moreover reports from the media and staff on the ground stressed community resistance and fear. There were individuals with symptoms refusing to seek care in the formal health system and instead resorting to home treatments from local pharmacies or traditional healers. With fear, despair and death all around, the community reluctance moved to an extreme level with riots and killings which started in April 2014, less than one month after the official confirmation of the epidemic. By September, eight people, including journalists, Ebola-related educators and health technicians had been killed in Guinea [1]. By early December community resistance had been reported in 9 of the 17 affected districts [6].

Many experts have attempted to explain the reasons behind the community resistance but most analysis was based on comments, testimonies and observations [1, 5]. Community members themselves were rarely consulted to understand their fears and perceptions for appropriate and urgent actions to be taken.

It is widely acknowledged that understanding and addressing communities’ needs and working side by side with local populations can significantly impact disease burden in affected communities. To support the efforts of the government and other institutions and organisations engaged in the fight against the expanding Ebola virus epidemic in Guinea, and to define appropriate strategic measures as part of a long term programme of health system strengthening, Amref Health Africa initiated a rapid assessment in selected sites at the peripheral level of the health system in their supported communities to identify gaps in Ebola response actions and understand the root causes for failure. This paper reports the main challenges faced by frontline health services and communities, and explores the perceptions and views on the current Ebola response in the Prefectures of Coyah and Forecariah in Guinea.

## Methods

To describe and explain the various interventions to the outbreak and the collective and respective impact of each on the epidemiological, clinical, economic, social, cultural and, to an extent, political dynamics, we conducted an analytical review of all scientific and media publications related to the EVD epidemic.

## Current status of knowledge

### Donations and spending to ease economic impact

Having been in a period of peace and economic recovery, Liberia, the Republic of Guinea (Guinea-Conakry) and Sierra Leone have since the onset of the EVD outbreak experienced reversal of development gains. The actual growth for 2014 for the three countries fell short of the projected growth figures from before the EVD epidemic [3] and this pattern continues for 2015 (Table 1).

**Table 1:** World Bank projected growth figures before and during the EVD epidemic

Country	Projected Growth for 2014 in June 2014 (%)	Projected growth for 2014 in December 2014 (%)	Projected growth for 2015 in June 2014 (%)	Projected growth for 2015 in December 2014 (%)	Cost estimated for 2015 (million US $)	Equivalent percentage of GDP (%) for cost estimated for 2015
Liberia	5.9	2.2	6.8	3.0	234	12
Sierra Leone	11.3	4.0	8.9	-2.0	439	8.9
Guinea	4.5	0.5	4.3	-0.2	142	2.3

In addition, the total fiscal impact of EVD is more than US$500million in 2014 alone, imposing additional budgetary needs of more than 6.0 percent of GDP in Liberia, more than 3.0 percent in Guinea, and more than 2.5 percent in Sierra Leone [3]. Further costs are estimated for 2015 with the continuing epidemic (Table 1). Public investment cuts have reached more than $160 million over all three countries as a result of the public diverting spending towards tackling the EVD outbreak. This necessary action further hampers the future economic prospects of West Africa [3, 4]. To help quell the epidemic and ease the financial burden, several nations and organizations pledged huge donations [5] and increased spending in West Africa. Projected growth for Liberia increased by 1% in November 2014 compared to that of October [5]. This increase correlates to evidence of the Ebola epidemic abating and economic activity increasing [5, 6]. However, overall donations fell significantly short of the needs to control the outbreak thereby hindering the efforts of local and foreign aid workers [7].

### Combating the lack of Infrastructure in West Africa

Guinea, Liberia and Sierra Leone all have very weak health systems with lack of adequate infrastructure and qualified personnel [8]. Table 2 shows the disproportion ratio of health personnel per 10,000 population in Liberia, Sierra Leone and Guinea Conakry in the years preceding the epidemic. Unsurprisingly, an outbreak of Ebola in densely populated areas in these countries with cases spanning wide geographic dispersions and complicated with public misconceptions, anxiety and fear needed immediate and effective response. However, there were significant failures in response during the early disease outbreak stage when the outbreak was assumed to be “comparable” to previous African outbreaks [9] and traditional containment methods were applied. Owing to lack of beds, more than half the cases of EVD cases in Sierra Leone were not hospitalized [10]. Local staff inefficiencies in dealing with EVD [11], due to the lack of training and experience, resulted in more widespread transmission especially among health care providers [12]. Supplies of personal protective equipment (PPE) often were inadequate, and where available improperly used [12]. Training programs for healthcare personnel working in the epidemic [13], which often included simulation exercises using Ebola survivors [14] have helped control spread and curb the epidemic. A WHO-led Ebola training facility, run by British military personnel, trains 120 health workers a week in Sierra Leone [12]. Confirmed EVD diagnosis requires real time polymerase chain reaction (RT-PCR) in a category 4 laboratory [15] by specially trained staff. The few laboratories in West Africa equipped to do this test were far from the Ebola hot zones and treatment centers. As a result, it can take several days to obtain confirmatory results. To make up for this, mobile diagnostic laboratories were donated and set up close to the Ebola treatment centres [16, 17]. In recent times, the WHO has approved a new ReEBOV Antigen Rapid Test Kit, which will decrease the time to diagnose EVD with acceptable specificity and sensitivity, and overcome many of the challenges associated with laboratory confirmation of EVD in the West African setting [18]. The international community has become more aware of the lack of infrastructure in West Africa and how devastating this can be in disease outbreaks. Developing the healthcare systems in West Africa may become more of a priority and awareness raised during this epidemic may initiate funding for this. However the affected countries have relied greatly on foreign aid. There is a need for more permanent measures, such as building hospitals, training programs, and more reliable and readily available funding schemes which will increase the capacity to quell outbreaks more rapidly and independently as seen in Nigeria and Senegal. Measures to reduce the frequency of zoonotic disease spread, such as regulation of bush meat markets, could also help. It is hoped that consistent aid investments in the health system will help prevent further crises and avert the spending of millions more in emergency aid down the line [19]. The EVD epidemic has made it possible to evaluate the success of different response methods in use. Current methods of preventing transmission, of diagnosing and treating patients, and of communication during outbreaks can all be improved. Lessons learnt from this outbreak and data gathered can be used to improve mathematical models, which can help guide the number and sizes of equipment, staff or treatment units needed as well as make sensible case and cost projections [20]. The UK for International Development and Wellcome Trust created a £6.5million research initiative to fund EVD-related research programs with an aim to better understand the management of Ebola outbreaks [12].

**Table 2:** Ratio of health personnel to population in Liberia, Sierra Leone and Guinea Conakry – World Health Statistics 2010

Country	Number of Physicians per 10,000 population (from year 2000-2009)	Number of Nurses or Midwifery staff per 10,000 population (from year 2000-2009)	Number of hospital beds per 10,000 population (from year 2000-2009)	Health expenditure per capita at average exchange rate (US $ in 2007)
Liberia	<0.5	3	7	22
Guinea	1	<0.5	3	26
Sierra Leone	<0.5	2	4	14
UK	21	6	39	3867
US	-	98	31	-
Global	14	28	27	802

### Raising awareness in West Africa

Having never encountered EVD before, many people in West Africa did not understand why they had to adapt their routine daily practice, while not enough people sought medical care in hospitals. There was an outburst of false rumors and violent attacks [21] as a result of fear and mistrust of foreign aid workers. Potentially contaminated equipment were looted, and sick relatives hidden [22]. A mob attack at an Ebola Treatment clinic in Liberia in August 2014 caused 17 Ebola patients to flee. Good communication to the areas affected is necessary to raise awareness of the dangers of EVD, educate the masses on modes of transmission and the need to limit or change certain practices, and encourage the need to seek treatment quickly. Many communication methods have been used to raise awareness in West Africa, including the Trilogy Emergency Relief Application (TERA) system, which sent out about 2 million mobile phone text messages a month in Sierra Leone [23]. However, it has been argued that the communication caused a great deal of fear, which some believe was unnecessary given that EVD accounts for only a small proportion of deaths in Africa compared to AIDS, diarrheal diseases and respiratory infections [24]. The Liberian president, Mrs. Ellen Johnson Sirleaf, said that the messages provided were “contrary to local tradition and culture” [25]. A justified response may be to invest into appropriate and culturally sensitive mediums of communication. Greater involvement from religious and political authorities in raising awareness of Ebola and combatting fear is crucial, and the WHO identified community participation as a critical success factor for Ebola control [21].

### Raising awareness globally

Public awareness, border screening, healthcare worker training and setting up isolation facilities are all crucial in the identification and early diagnosis of imported EVD cases [26]. EVD has become a dominant headline across all forms of media; with the word “Ebola” being the most commonly used keyword across US, international and African publications between 3rd August and 8th August 2014 [27]. Despite only one American death between September 1st and October 31st 2014, the word “Ebola” received 10 times more mentions in US online media compared to heart disease which killed more than half a million Americans during the same period [28]. Unfortunately, the mass media response caused a great deal of fear. Tom Frieden, the Director of the Centres for Disease Control and Prevention (CDC) believes that the media coverage may “exaggerate the potential risks” of EVD. Certain aid agencies claimed they were “facing an epidemic of magnitude never seen before” which some felt was fear mongering [25]. Ebola, which has an R0 value of approximately 2, is far less contagious compared to other infectious diseases, such as measles with an R0 of 17 [20]. Many reason that the exaggerated coverage has led to EVD becoming one of American's top health worries with approximately one in six naming Ebola as the nation's top health concern [29]. Nonetheless, it can be argued that the benefits of public sensitization outweigh the potential harms. Dr Anthony S. Fauci, Director of the US National Institute of Allergy and Infectious Disease, said “the publicity associated with (Ebola) is a good thing even though it may be scaring some people, because it is making people aware” [30].

### Preventing spread of Ebola

Without the current intervention, the CDC estimated that the number of EVD cases in Liberia and Sierra Leone alone could rise to between 550,000 and 1.4 million in 2015 [31]. There have been over 24,282 confirmed, probable and suspected cases and 9,976 reported deaths in Liberia, Guinea and Sierra Leone to March 2015 [32]. In an attempt to prevent global spread, many airlines suspended flights to and from some West African countries [3, 33] despite discouragement from the WHO. Only 35 cases have been reported outside the main transmission zone [32] despite increased global travel nowadays and these cases have been manageable using strict infection control measures [4, 34] in countries with strong health care systems. However, the travel restrictions have negatively affected tourism, import of airfreight, and delivery of essential medical supplies and health experts to the affected areas. Liberia had its incoming flights reduced from 27 per week to just 6 per week [5]. Prevening mass gatherings and closing schools and places of work have been employed to prevent spread locally. Evidence suggests that the number of cases have been lower than predictions in some places. For example, the 20,000 cumulative cases predicted by November 2014 in Nigeria were not reached and therefore the conditions of the epidemic changed due to appropriate response. Furthermore, the incidence rate has seen an increase from 12-17 days doubling time to 15-30 days [10]. However, these control measures may not have been justified methods and may have aggravated fear and economic loss. “The ‘tide of fear’ triggered by the outbreak could cause 80% to 90% of the economic impact” said Jim Yom Kim, the World Bank President. The fear factor from the Ebola outbreak reduced labor force participation, closed places of employment and disrupted transportation [35]. Local trade has also been affected as EVD survivors are shunned [36]. The WHO estimated that the 2-year regional financial impact range from US$3.8 billion to US$32.6 billion [5]. The outbreak led to the suspension of some non-Ebola aid work [37] and the closure of some hospitals [19] leaving non-EVD patients in need of medical aid helpless [15]. ‘Symptoms of racism’ triggered out of fear of contact with individuals from West Africa have been reported. A notable case was that of two students from Nigeria denied admission to a Texas college on the basis that the college was no longer accepting applications from countries with confirmed Ebola cases, despite the WHO declaring Nigeria as Ebola free [38]. The WHO failed to act quickly enough to quell the outbreak [11]. The presence of EVD was not confirmed until 3 months after the index case [25] and it took 5 months and 1000 deaths before a public health emergency was declared by WHO [17]. Furthermore, Ebola-like symptoms were documented in Sierra Leone in March 2014, but the official recognition of the spread to Sierra Leone did not arrive until May 2014. It was felt that “modest further intervention efforts” early on could have brought the outbreak under control [25]. The epidemic has not yet finally abated meaning that there is ever more potential for viral evolution as the virus spreads. At least 341 mutations in EVD have occurred since late August and the higher the case count, the more chance for another mutation to occur and the greater possibility of the virus [20]. This could make it more devastating and also render the current candidate vaccines ineffective [39].

### Treating EVD

High CFRs and a great deal of suffering are caused by EVD. The average EVD CFR is around 50% but up to a 90% CFR has been recorded when no treatment is provided [40]. Hydration and supportive therapy are known to lower infection and mortality. It is thought that the therapy allows enough time for the immune system to combat the virus [41]. Despite its effectiveness, many patients may have missed out on treatments such as rehydration therapy, because of the “inaccurate view” that there is no proven Ebola treatment [42]. Furthermore, Dr. Hoolihan of Save the Children said that the process of giving intensive fluids intravenously can only happen “when training and facilities are in place”, because the use of needles can put health workers at risk. The accelerated production of experimental drugs and vaccines has proved justified so far. The drug z-Mapp has been shown to reduce the viral load in macaques to an undetectable level after 21 days and has been used successfully at the Royal Free Hospital when treating an EVD patient. Another drug called Favipravir has already been safety tested and is being investigated as a possible treatment [43], as well as others such as brincidofovir and TKM-Ebola [44]. Although no vaccine has been put into use, phase 1 safety trials are underway for cAd3-ZEBOV and rVSV-ZEBOV, the two current candidate vaccines, with promising results [15, 45]. It is hoped that these will improve response to future outbreaks as well as reduce the risk of epidemics.

## Conclusion

It can be concluded that the overall response has been justified despite some failures. Donations have helped relieve some of the financial pressure in West Africa, improvements to the infrastructure and staffing are being made, awareness has been raised and little spread to the rest of the world has been noted. The drugs and vaccines developed could also be justified, but that is not clear yet. Although there has been a great amount of fear caused, which in turn has consequences, and not all methods used have been effective or appropriate, there are lessons to be learnt with reasons to improve response for future disease outbreaks. Importantly, as far as Ebola treatment is concerned, the medical community has learned what is effective in the simple therapeutic compendium, such as aggressive rehydration of patients.
